# Strain in perovskite solar cells: origins, impacts and regulation

**DOI:** 10.1093/nsr/nwab047

**Published:** 2021-03-23

**Authors:** Jinpeng Wu, Shun-Chang Liu, Zongbao Li, Shuo Wang, Ding-Jiang Xue, Yuan Lin, Jin-Song Hu

**Affiliations:** Beijing National Laboratory for Molecular Sciences (BNLMS), Institute of Chemistry, Chinese Academy of Sciences, Beijing 100190, China; School of Chemical Sciences, University of Chinese Academy of Sciences, Beijing 100049, China; Beijing National Laboratory for Molecular Sciences (BNLMS), Institute of Chemistry, Chinese Academy of Sciences, Beijing 100190, China; School of Chemical Sciences, University of Chinese Academy of Sciences, Beijing 100049, China; School of Material and Chemical Engineering, Tongren University, Tongren 554300, China; Beijing National Laboratory for Molecular Sciences (BNLMS), Institute of Chemistry, Chinese Academy of Sciences, Beijing 100190, China; Beijing National Laboratory for Molecular Sciences (BNLMS), Institute of Chemistry, Chinese Academy of Sciences, Beijing 100190, China; School of Chemical Sciences, University of Chinese Academy of Sciences, Beijing 100049, China; Beijing National Laboratory for Molecular Sciences (BNLMS), Institute of Chemistry, Chinese Academy of Sciences, Beijing 100190, China; School of Chemical Sciences, University of Chinese Academy of Sciences, Beijing 100049, China; Beijing National Laboratory for Molecular Sciences (BNLMS), Institute of Chemistry, Chinese Academy of Sciences, Beijing 100190, China; School of Chemical Sciences, University of Chinese Academy of Sciences, Beijing 100049, China

**Keywords:** perovskite, stress, strain, stability, solar cells

## Abstract

Metal halide perovskite solar cells (PSCs) have seen an extremely rapid rise in power conversion efficiencies in the past few years. However, the commercialization of this class of emerging materials still faces serious challenges, one of which is the instability against external stimuli such as moisture, heat and irradiation. Much focus has deservedly been placed on understanding the different origins of intrinsic instability and thereby enhancing their stability. Among these, tensile strain in perovskite films is an important source of instability that cannot be overcome using conventionally extrinsic stabilization approaches such as encapsulation. Here we review recent progress in the understanding of the origin of strain in perovskites as well as its corresponding characterization methods, and their impacts on the physical properties of perovskites and the performance of PSCs including efficiency and stability. We then summarize the latest advances in strain-regulation strategies that improve the intrinsic stability of perovskites and photovoltaic devices. Finally, we provide a perspective on how to make further progress in stable and high-efficiency PSCs via strain engineering.

## Teaser

This review summarizes the origins, impacts and regulation methods of strain in perovskites, and provides a perspective on the further development of strain engineering in perovskites.

## INTRODUCTION

The power conversion efficiencies (PCEs) of perovskite solar cells (PSCs) have improved rapidly from 3.8% to a certified 25.2% for single junction devices and approaching 30% for perovskite-based tandem devices in the past few years [1–6]. Such excellent performance can be mainly attributed to their long carrier diffusion lengths and low trap densities arising from their defect-tolerant properties [7–10]. However, the instability of PSCs remains the largest barrier toward their commercialization [11–14]. Lead halide perovskites have been reported to be sensitive to many external stimuli, such as moisture, oxygen, heat and ultraviolet light [15,16]. Recent efforts have demonstrated progress in enhancing the stability of PSCs extrinsically by introducing hydrophobic coating, replacing reactive metal electrodes with non-corrosive carbon or transparent conducting oxides, and encapsulation techniques [17–20]; these approaches straightforwardly protect perovskite devices from the ambient environment. Once the degradation of PSCs induced by external stimuli is suppressed, the intrinsic instability of perovskite material itself—which cannot be addressed using extrinsic protection methods—would impose a limitation on the stability of whole devices [21].

Perovskites, especially the organic-inorganic hybrid perovskites, possess a flexible crystal structure owing to their soft lattice. This enables the manipulation of structural and optoelectronic properties of perovskites through strain engineering [22–25]. For instance, by applying appropriate hydrostatic pressure, the bandgap of CH_3_NH_3_PbI_3_ becomes narrower that broadens the absorption spectrum of this material, while the carrier lifetime is prolonged, both of which are beneficial for achieving better photovoltaic (PV) performance [26–30]. When further applying pressure as high as 60 GPa, CH_3_NH_3_PbI_3_ exhibits metallic character through apparent bandgap closure. This pressure-induced semiconductor-to-metal transition demonstrates the realization of wholly new electronic structure and transport properties in perovskites, greatly enriching the electronic diversity of perovskites [31,32]. Nevertheless, strain, especially the tensile strain in perovskite, is known to contribute to instability in these materials; this weakens bonds, favors the formation of defects and lowers the activation energy for ion migration, thereby accelerating the degradation of perovskites [31,33,34]. Tensile-strain-induced intrinsic instability is now widely identified as a major bottleneck toward the achievement of stable PSCs [21,35].

**Figure 1.**
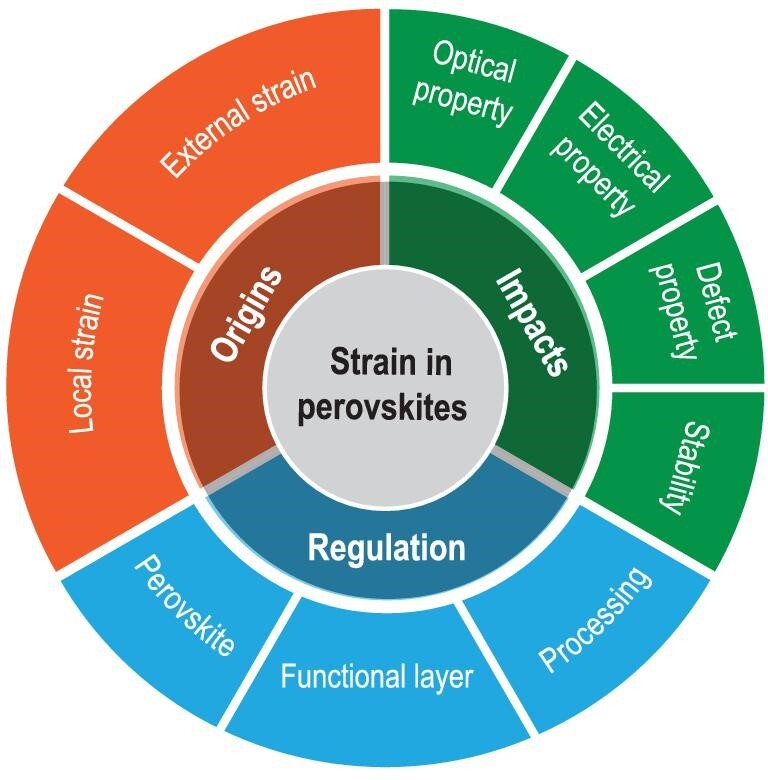
Strain origins, impacts and regulation strategies discussed in this review.

Here we review the latest advances that correlate the strain in perovskites with their physical properties and PV performance including efficiency and stability, and discuss the avenues for further progress towards high-efficiency and stable PSCs via strain regulation (Fig. 1). We begin with a discussion of two different origins of strain/stress in perovskites: (i) local lattice strain, which originates from the ionic size mismatch between the A cation and the lead halide cage size in ABX_3_, and the local lattice mismatches due to the inhomogeneity in mixed halide perovskites; (ii) external condition-induced strain, including thermal expansion mismatch between perovskites and the contacting functional layers, and lattice mismatch between perovskites and epitaxial substrates. We then discuss the characterization of strain, and its impact on the physical properties of perovskites (electronic structure, ion migration and defect) and the efficiency and stability of PSCs. We follow with a summary of the recent advances in strain-regulation methods that release the tensile strain and thereby improve the intrinsic stability of perovskites and corresponding PV devices. Finally, we provide a perspective on further strain-engineering innovations for high-efficiency and stable PSCs.

## ORIGINS OF STRAIN IN PEROVSKITES

Since the first finding of residual strain in perovskites reported in 2015, much concern has been raised regarding the strain-induced issues of PSCs, especially the material and device instability [36]. Understanding the origin of strain would therefore be an essential precondition to fabricating stable PSCs [21]. In this section, we provide a brief review on the origins of strain in perovskite, excluding conventional strain-engineering methods such as hydrostatic pressurization and bending the flexible substrates in either a concave or convex shape. There are generally two different origins of strain in perovskites: local lattice strain and external condition-induced strain.

### Local lattice strain

**Figure 2.**
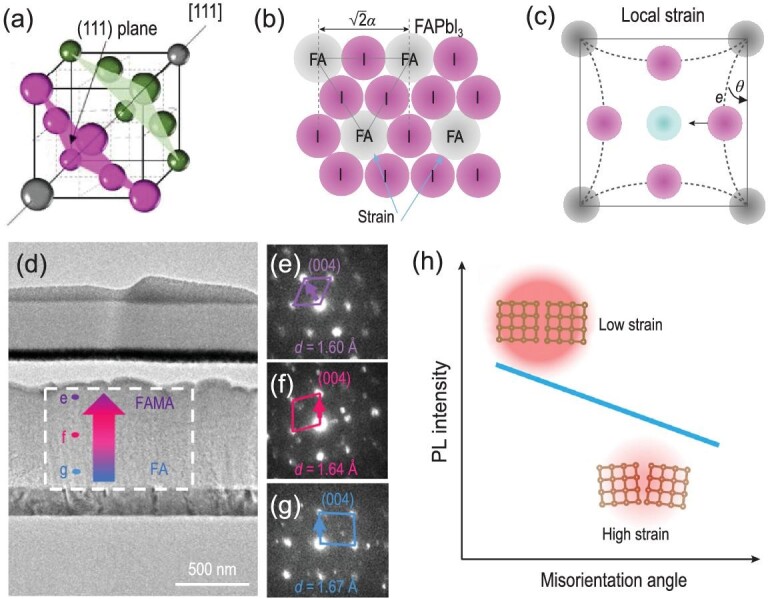
(a) Crystal structure of perovskite in cubic close packing model. (b) A close-packed layer in FAPbI_3_, displaying the structural strain. (c) Schematic illustration of the local strain [51]. Copyright 2018, Springer Nature. (d) The cross-sectional transmission electron microscopy (TEM) image of (FAPbI_3_)_0.85_(MAPbBr_3_)_0.15_-based solar cell. (e–g) The nano-beam electron diffraction patterns ([100] zone axis at point e, f and g in (d)) [35]. Copyright 2019, Springer Nature. (h) Correlation between misorientation angle and degrees of strain [52]. Copyright 2019, Elsevier.

Metal halide perovskites have the general formula ABX_3_ [37,38], where A is a monovalent cation, such as organic methylammonium (MA, CH_3_NH_3_^+^) [39,40] or formamidinium (FA, (NH_2_)_2_CH^+^) [7,41], and inorganic cesium or rubidium ion; B is a divalent metal cation, including Pb^2+^, Sn^2+^ or Ge^2+^; and X is a mixture of halogen anion (Cl^−^, Br^−^ and I^−^) [42–47]. The stable crystal structures of perovskites can be predicted by a reliable empirical index, Goldschmidt tolerance factor (*t*). This is calculated from the ionic radius of the atoms as follows: *t* = (R_A_ + R_X_)/[$\sqrt 2 $(R_B_ + R_X_)], where R_A_, R_B_ and R_X_ are the ionic radii of the corresponding ions. Metal halide perovskites tend to form an ideal cubic structure when 0.8 < t < 1, an orthorhombic structure when t < 0.8 and a hexagonal structure when t > 1 [48–50]. Besides structural stability, the size of ions also affects the stability of perovskites in ambient air [7]. Sargent *et al.* [51] revealed that mixed CsMAFA perovskites showed better stability than FAPbI_3_. They found that this instability of FAPbI_3_ was attributed to the local lattice strain. This strain arises from the ionic size mismatch between the FA cation and the lead iodide cage size owing to the large size of the FA cation, leading to cage distortions and PbI_6_ octahedra tilting (Fig. 2a–c). Therefore, a perovskite structure with ionic size mismatch would tend to form local lattice strain.

Local lattice mismatch is another origin of local lattice strain in perovskites, especially the mixed halide perovskites, despite the fact that the highest PCEs of PSCs are mostly achieved by employing these perovskites. Mixed halide perovskites have been known to suffer from materials inhomogeneity due to composition separation into separated Br-rich and I-rich phases when exposed to heat and light. This is attributed to the substantial chemical mismatch among each component, and the unbalanced growth conditions of each component during the process of film fabrication. Recently, Chen *et al.* [35] revealed that this inhomogeneity in perovskite films perpendicular to the substrate resulted in the local lattice mismatch, then the lattice distortion of microscopic crystal structure, and consequently the local lattice strain (Fig. 2d–g), which may be a type of microstrain. Besides local lattice mismatch, local crystal misorientation can lead to local strain within perovskite grains. The grain-to-grain orientation spread further results in local strain heterogeneity within halide perovskite films (Fig. S1a). Ginger *et al.* [52] have detailed this origin of local strain through ultrasensitive electron backscatter diffraction (EBSD), which can reveal crystal orientation variations in a sub-grain structure. They demonstrated the correlation between misorientation-induced local strain and misorientation angles obtained by EBSD and found that the larger misorientation angle led to higher degrees of strain (Fig. 2h).

### External condition-induced strain

**Figure 3.**
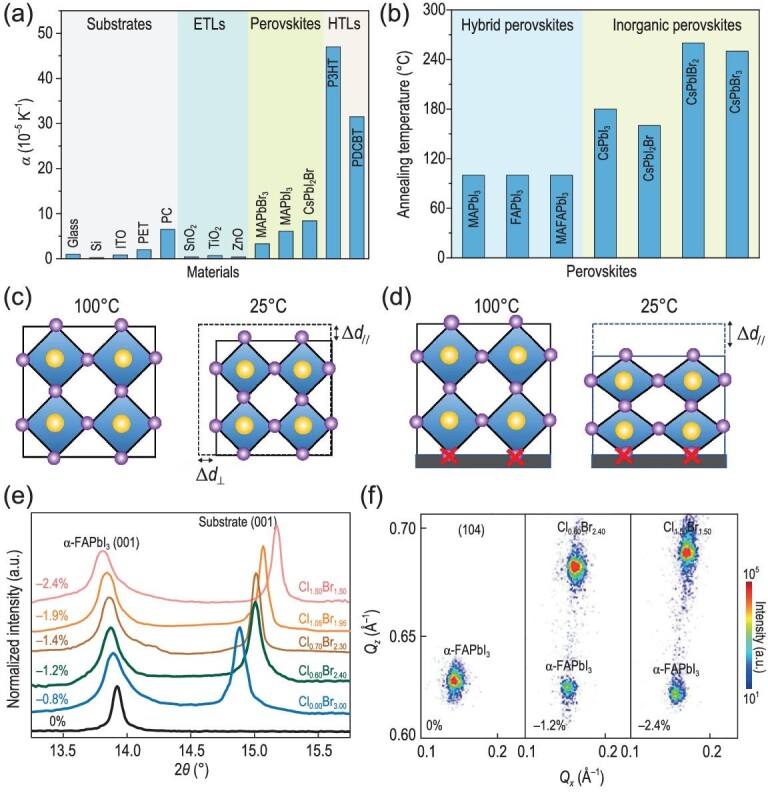
(a) Thermal expansion coefficients of functional layers in PSCs including perovskites, substrates, ETLs and HTLs. (b) Different annealing temperatures of perovskites [57]. Copyright 2020, Springer Nature. (c) Schematic illustration of the contraction of perovskite film without substrate during cooling. (d) Schematic illustration of the formation of tensile strain in perovskite film with substrate adhesion during cooling [34]. Copyright 2017, The American Association for the Advancement of Science. (e) High-resolution XRD patterns of the (001) peaks of the epitaxial films on different substrates. (f) Reciprocal space mapping with (104) asymmetric reflection of the α-FAPbI_3_ films [58]. Copyright 2020, Springer Nature.

The external condition-induced strain originates from two types of mismatches: thermal expansion and lattice mismatch between the perovskite and substrate, which may be assigned to biaxial strain. Figure 3a summarizes the linear thermal expansion coefficients (α) of lead halide perovskites, substrates and other functional layers including electron-transport layers (ETLs) and hole-transport layers (HTLs). Perovskites possess high values of α ranging from 3.3 to 8.4 × 10^−5^ K^−1^ that correspond to the volumetric thermal expansion coefficient (α_v_) of cubic perovskites ranging from 9.9 to 25.2 × 10^−5^ K^−1^, larger than Cu(In,Ga)Se_2_ (α_v_ = 2.7 × 10^−5^ K^−1^) [53] and CdTe (α_v_ = 1.4 × 10^−5^ K^−1^) [54]. The widely used ITO-coated glass and metal oxide charge transport layers have much lower α values in the range of 0.37 to 1 × 10^−5^ K^−1^ [55,56]. Therefore, there is a large thermal expansion difference (Δα) between the perovskite and substrate.

To achieve high-efficiency PSCs, the perovskite films typically require annealing at high temperature >100°C to enhance the crystallinity and reduce defects. Figure 3b shows the processing temperatures of different kinds of perovskites including hybrid organic-inorganic perovskites and all-inorganic perovskites [57]. It should be noted that all-inorganic perovskites need even higher temperatures to form the black cubic perovskite phase compared with hybrid organic-inorganic perovskites. For example, CsPbI_3_ requires annealing temperatures in the range of 180 to 330°C. There would be a large temperature gradient (ΔT) when perovskite films cool from the annealing temperature to room temperature [31].

The large Δα and ΔT thus lead to the thermally induced strain. This can be quantified using the following equation:

(1) \begin{equation*}{\sigma _{\Delta T}} = \frac{{{E_P}}}{{1 - {v_P}}}\left( {{\alpha _S} - {\alpha _P}} \right)\!\Delta T\ ,\end{equation*}

where *E_P_* is the modulus of perovskite, ν*_P_* is the Poisson's ratio in perovskite, and α*_P_* and α*_S_* are the thermal expansion coefficients of perovskite and substrate, respectively. In particular, when a perovskite film forming at high temperature cools to room temperature, it would contract owing to the positive thermal expansion coefficient (Fig. 3c). If a perovskite film is deposited on a substrate with lower α, the contact formed between perovskite and substrate during the high-temperature annealing process constrains the perovskite from contracting when it cools back to room temperature, leading to tensile strain along the in-plane direction (Fig. 3d). A compressive strain is simultaneously formed in the out-of-plane direction in view of the positive Poisson's ratio in perovskites.

Another origin of external condition-induced strain is the lattice mismatch between the perovskite and epitaxial substrate. Xu *et al.* [58] reported the strained epitaxial growth of α-FAPbI_3_ single-crystal thin films on lattice-mismatched halide perovskite substrates. They first tuned the substrate composition of MAPbCl_x_Br_3−x_, providing a series of host substrates with different lattice parameters. α-FAPbI_3_ was then epitaxially grown on such substrates by the inverse temperature growth method. Figure S1b shows the optical images of the as-grown epitaxial α-FAPbI_3_ film. From the high-resolution X-ray diffraction (XRD) patterns of these epilayers (Fig. 3e), the substrate MAPbCl_x_Br_3−x_ peak shifts to higher diffraction angles as x increases, while the α-FAPbI_3_ peak shifts to lower diffraction angles [59,60]. Such a shifts to lower angles indicates the increase of the out-of-plane lattice parameter, demonstrating the decrease of the in-plane lattice parameter and then the increase of in-plane compressive strain. When x is above 1.5, the corresponding strain level of the α-FAPbI_3_ film is calculated to be as high as −2.4%. The reciprocal space mapping of strain-free and strained α-FAPbI_3_ films with different lattice mismatch with the substrate (Fig. 3f) further indicates the formation of strain via the lattice mismatch between the perovskite and epitaxial substrate [61].

## CHARACTERIZATION OF STRAIN IN PEROVSKITES

Since strain directly alerts the lattice parameters of perovskites, XRD is thereby a convenient and effective technique to measure the strain in perovskites [34]. The strain can be calculated from the shift of XRD peaks:

(2) \begin{equation*}\varepsilon = \ \frac{{{d_{\mathit {strained}( \mathit {hkl} )}} - {d_{\mathit{non\hbox{-}strained}( {\mathit {hkl}} )}}}}{{{d_{\mathit {non\hbox{-}strained} ( {\mathit {hkl}} )}}}},\end{equation*}

where *d*_*strained(hkl)*_ and *d*_*non-strained(hkl)*_ are the crystal plane spacing of perovskites with and without strain, respectively. The non-strained crystal plane spacing can be obtained from the freestanding perovskite powders prepared by scraping the as-prepared perovskite films from substrates. According to the positive Poisson's ratio in perovskites, if there is a tensile strain in perovskite film along the in-plane direction, the spacing of crystal planes along this direction becomes smaller; the direction perpendicular to the substrate would be under compressive strain simultaneously, leading to the larger plane spacing along this direction, and vice versa.

**Figure 4.**
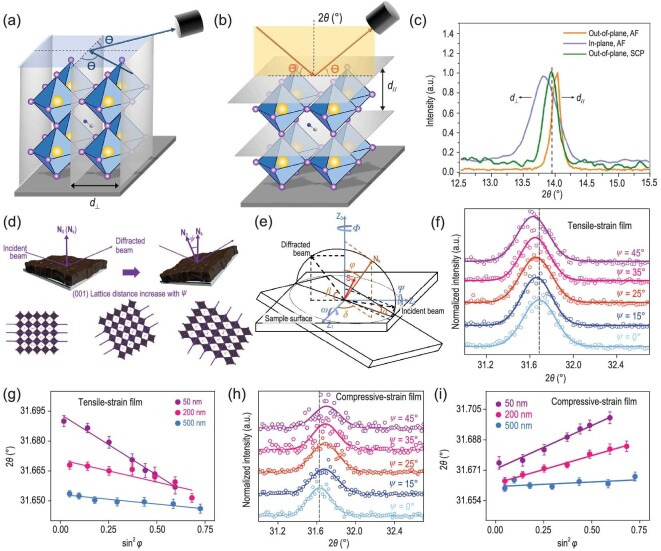
Schematics of (a) out-of-plane and (b) in-plane XRD. (c) In-plane and out-of-plane XRD of AF and out-of-plane SCP [34]. Copyright 2017, The American Association for the Advancement of Science. (d) Schematic of the strain measurement using GIXRD, where N_0_ is the sample normal direction, N_k_ is the diffraction vector and ψ is the instrument tilt angle. (e) Diffraction geometries of depth-dependent strain distribution measurement. GIXRD spectra at different tilt angles at a depth of 50 nm for (f) tensile-strained film and (h) compressive-strained film. Strain distribution at depths of 50, 200 and 500 nm as a function of sin^2^ϕ for the (g) tensile-strained and (i) compressive-strained film. The error bar represents the standard deviation of 2θ [35]. Copyright 2019, Springer Nature.

There are two different XRD modes: in-plane and out-of-plane measurements. The former measures the plane spacing of the planes parallel to the substrate, while the latter characterizes the plane spacing perpendicular to the substrate, as illustrated in Fig. 4a and b. Micro XRD can further characterize the strain variations on a range of length scales, which can investigate the microstructural phase in very small analysis regions ∼0.11 mm in dimension [62]. Huang *et al.* [34] compared the in-plane and out-of-plane XRD of annealed MAPbI_3_ film (AF) and out-of-plane XRD of scraped MAPbI_3_ power (SCP) from annealed film (Fig. 4c). The out-of-plane XRD peak of AF shifted to a higher diffraction angle compared to that of strain-free SCP. Such a shift demonstrated the smaller plane spacing in the direction perpendicular to the substrate, indicating the compressive strain along this direction. As expected, the in-plane XRD peak of AF shifted to a lower diffraction angle compared with the out-of-plane XRD peak of AF, determining the larger plane spacing and thus indicating the tensile strain in the in-plane direction of the film.

Grazing incident XRD (GIXRD) has proven to be a powerful technique for characterizing the structural depth profiling of films. This can further be used to study the depth-dependent strain distribution in perovskite films. Specifically, the information from different depths from the surface to bulk is obtained by changing the incident angel; the larger the incident angle, the deeper X-ray penetrates from the surface. Chen *et al.* [35] recently performed GIXRD to investigate the evolution of in-plane residual strain over the film thickness in mixed perovskite (FAPbI_3_)_0.85_(MAPbBr_3_)_0.15_ films, wherein the classical sin^2^ϕ measurement is united with GIXRD (Fig. 4d and e). They fixed the 2θ and varied the instrument tilt angle ψ to obtain corresponding XRD patterns (Fig. 4f). There exists a systematic shift in peak position to lower 2θ as the penetration depth increases. The slope of the fitting line from the linear relationship between sin^2^ϕ and 2θ further indicates the magnitude of strain (Fig. 4g). The above results demonstrate the gradient distribution of tensile strain in the perovskite thin film, wherein the tensile strain gradually decreases from the top surface to the bulk of the film. This method is also applicable to the characterization of compressive strain in perovskite films, as shown in Fig. 4h and i.

Considering the long-term X-ray radiation-induced phase segregation, best practice with regard to strain determination by XRD techniques could consist of the following two methods: (i) choosing single halide perovskites may be better than mixed halide perovskites, whereas phase segregation induced by other factors such as doping and boundary effect under light irradiation may also influence the accuracy of strain characterization; (ii) a combination of in-plane and out-of-plane XRD measurements. Based on the positive Poisson's ratio in perovskites, if one direction has tensile strain, the vertical direction would have compressive strain, and vice versa. The shift of in-plane XRD peaks therefore is opposite to that of out-of-plane XRD peaks. If there is just a composition-induced peak shift rather than strain-induced shift, the in-plane and out-of-plane XRD results are the same.

**Figure 5.**
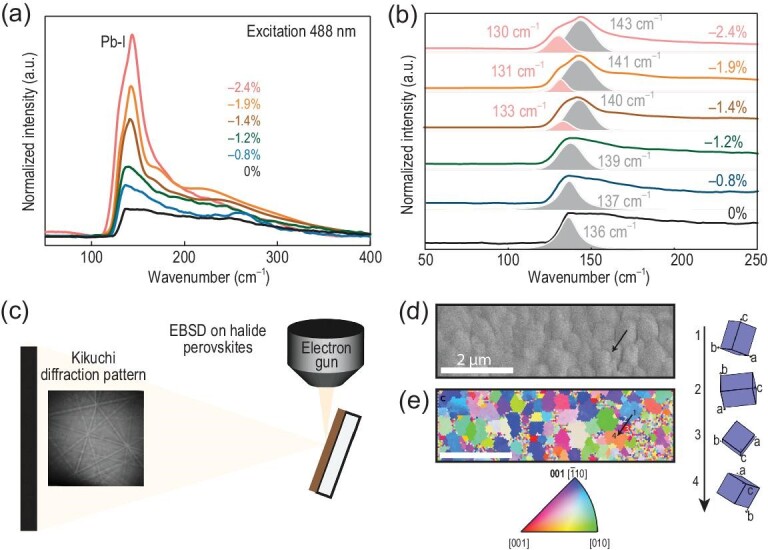
(a) Confocal Raman spectra of the epitaxial α-FAPbI_3_ layer at different strains. (b) Fitting analysis of the Raman peaks in (a) [58]. Copyright 2020, Springer Nature. (c) Schematics of EBSD measurement on CH_3_NH_3_PbI_3_ thin films. (d) SEM image and (e) inverse pole figure (IPF) map generated from EBSD of CH_3_NH_3_PbI_3_ thin film with IPF color key. The right column depicts the changes in local crystal orientation along the black arrow in (d) and (e) [52]. Copyright 2019, Elsevier.

Besides the quantitative XRD measurement, there are also other qualitative characterization methods for the measurement of strain in perovskites. Xu *et al.* [58] applied Raman spectroscopy to study the structure of α-FAPbI_3_ at different strains between 0% and −2.4%. The peak at 136 cm^−1^ began to split into two peaks at 140 (main) and 133 cm^−1^ (shoulder) (Fig. 5a and b). The two peaks shifted to 143 and 130 cm^−1^ as the strain was further increased to −2.4%. This blueshift of the main peak can be attributed to the compression in-plane Pb-I bond, while the redshift of the shoulder peak was due to the stretching of out-of-plane Pb-I bond. Raman spectroscopy measurement can therefore be used to reveal the strain in perovskite films. Ginger *et al.* [52] characterized the crystal misorientation-induced strain using EBSD (Fig. 5c). The measured local crystal misorientation confirmed the presence of local strain (Fig. 5d and e). The transmission electron microscopy (TEM) measurement is also used to observe the strain in perovskite films through the measurement of crystal plane distance as shown in Fig. 2d.

As discussed above, there are four widely used methods to characterize the strain in perovskites, including XRD, Raman spectroscopy, EBSD and TEM (Table 1). According to equation (2), XRD can provide quantitative measurement of strain through the measured crystal plane spacing. A high-resolution TEM image with micro-area diffraction pattern can also measure the crystal plane spacing and then quantify the strain, however it usually suffers from being time-consuming and an *ex situ* process. Raman spectroscopy is only a qualitative characterization method for strain measurement. However, the shifting/splitting/broadening of Raman peaks under different strain conditions can be used to investigate the origin of strain through the analysis of variation in bond rigidity. EBSD that reveals grain structure and internal misorientation in perovskite films can characterize the strain within grains and grain boundaries.

**Table 1.** Summary of characterization methods for strain in perovskites.

Characterization methods	Instrumental accuracy	Measurement criterion	Measurement condition	Reference
XRD	Quantitative	Crystalline plane spacing	*In situ*	[34,35,57]
Raman	Qualitative	Shifting/splitting/broadening of Raman peaks	*In situ*	[58]
EBSD	Qualitative	Orientation	*In situ*	[52]
TEM	Quantitative	Crystalline plane spacing	*Ex situ*	[35]

## IMPACTS OF STRAIN ON PEROVSKITES

As discussed in the section ‘Origins of Strain in Perovskites’, there are several types of strain in perovskites. Particularly, some strains are inevitably residual in perovskites during the film preparation process, such as thermal expansion and lattice mismatch-induced strains. In this section we briefly review the impacts of strain, including both compressive and tensile strain, on the physical properties (electronic band structure, electrical properties, defect properties and ion migration) and stability of perovskites.

### Electronic band structure

**Figure 6.**
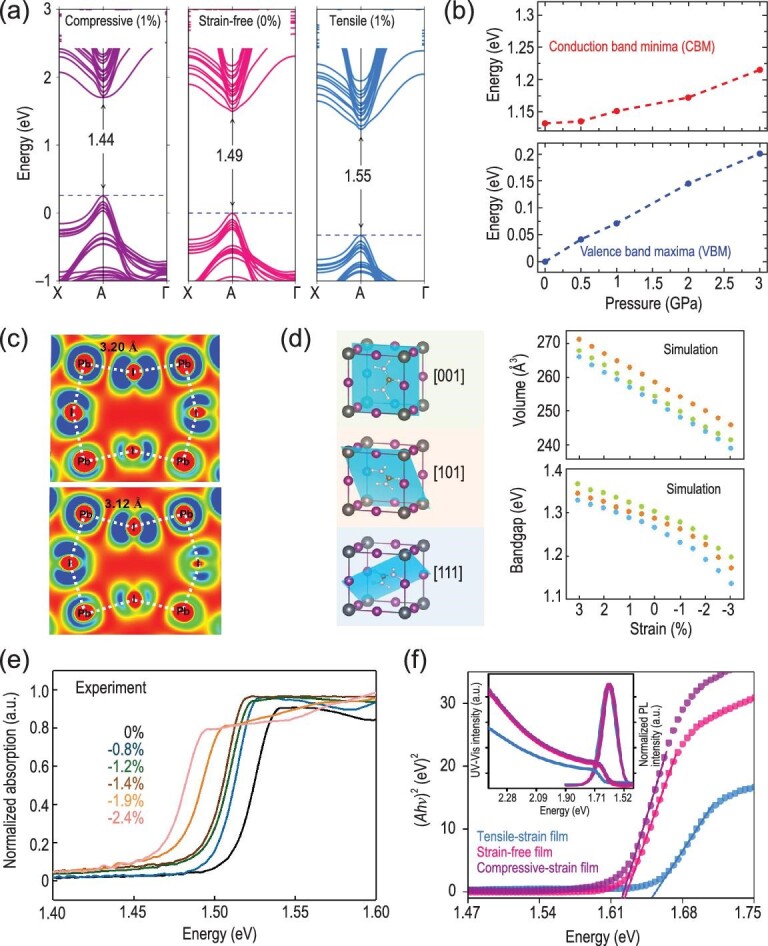
(a) Calculated strain-dependent band structures under biaxial tensile, zero and compressive strains from first-principle density functional theory-based approaches [35]. Copyright 2019, Springer Nature. (b) Modification in energy of CBM and VBM. (c) Electronic charge density of the VBM of FA_0.75_Cs_0.25_PbI_3_ under pressure of 0 GPa (upper panel) and 2 GPa (lower panel) [32]. Copyright 2019, The American Chemical Society. (d) Evolution of lattice volume and bandgap of three α-FAPbI_3_ lattices with different FA^+^ organic cation orientations as a function of strain. (e) Absorption spectra of α-FAPbI_3_ films under gradually increased compressive strains [58]. Copyright 2020, Springer Nature. (f) UV-Vis absorption spectra and photoluminescence (PL) spectra of (FAPbI_3_)_0.85_(MAPbBr_3_)_0.15_ under tensile-strain, strain-free and compressive-strain conditions [35]. Copyright 2019, Springer Nature.

Strain can lead to lattice distortion and further change the crystal structure of perovskites. This lattice deformation alters the electronic band structure. The calculated band structures of FAPbI_3_ under tensile, zero and compressive strains by Chen *et al.* [35] demonstrate that bandgaps show an increase as the strain changes from compression to tension (Fig. 6a). This also leads to the deeper defect levels of perovskites that may act as non-radiative recombination centers to lower the device performance with the precondition of defect energy levels being not sensitive to strain. Islam *et al.* [32] further performed high-level *ab initio* simulation techniques to investigate the compressive strain-induced changes in the electronic band structure of mixed-cation FA_0.75_Cs_0.25_PbI_3_. They found that the electronic band structure responds strongly to the compressive strain, wherein the compressed perovskites exhibit narrower bandgaps. These bandgap changes are mainly attributed to modulation of the valence band maximum (VBM) rather than conduction band minimum (CBM). As shown in Fig. 6b, the applied compressive pressure of 3 GPa lifts the VBM of FA_0.75_Cs_0.25_PbI_3_ upward by ∼0.2 eV; this strain pushes the CBM upward by only ∼0.08 eV. The feasible stress range for tuning the band structure may be under the compressive stress within 2 GPa, corresponding to the strain of 20%–13% considering the modulus of perovskites (10–15 GPa) [32]. This provides a controlled regulation of bandgap within 100 meV.

This different variation in band edges under strain can be explained through the charge densities of the VBM and CBM. It is well known that the VBM of perovskite consists mainly of the anti-bonding overlap between Pb 6s and I 5p orbitals, whereas non-bonding Pb 6p orbitals with a very small contribution from I 5p form the CBM. Under the compressive strain, the Pb-I bond lengths become shorter, leading to the tilting of the PbI_6_ octahedra (Fig. 6c). The shorter Pb-I bonds enhance the anti-bonding overlap between the Pb 6s and I 5p orbitals, thereby increasing the energy of the valence band edge. Although the tilting of the PbI_6_ octahedra lowers the Pb-I-Pb angles that reduce the anti-bonding coupling in the VBM, the shorter Pb-I bonds dominate over the distorted Pb-I-Pb angles, ultimately pushing the VBM upward. As for CBM, the non-bonding localized states of Pb 6p orbitals are less sensitive to the shorter Pb-I under compressive strain; there is only a little increase in the overlap between I 5p and Pb 6p non-bonding orbitals. The band edge of CBM therefore also shifts to higher energy but to a smaller extent compared to the VBM, consistent with the experimental results reported by Xu *et al.* [58] (Fig. S2a). Overall, the application of compressive strain shifts the VBM of perovskites such as α-FAPbI_3_ and FA_0.75_Cs_0.25_PbI_3_ to a higher energy that is beneficial for the better alignment between perovskite film and hole-transport layer including the widely used spiro-OMeTAD and PEDOT:PSS, and decreases the bandgap of perovskites.

The change of electronic band structures under strains then alters the bandgap of perovskites. Xu *et al.* [58] calculated the evolution of bandgap as a function of strain for three α-FAPbI_3_ lattices with different FA^+^ organic cation orientations (Fig. 6d). The bandgaps of α-FAPbI_3_ show redshift changes with applied strains from tension to compression. Figure 6e exhibits the absorption spectra of strained α-FAPbI_3_ thin films. The absorption onset redshifts with the increase of compressive strain. Photoluminescence (PL) spectra of α-FAPbI_3_ thin films under different strains from 0% to −2.4% further demonstrate the changes in bandgap (Fig. S2b). The PL peak shifts from ∼1.532 eV under no strain to ∼1.488 eV under the compressive strain of −2.4%, indicating a reduction of ∼35 meV in the bandgap and demonstrating the feasible strain range for tuning the bandgap of perovskites from experiment [58,63]. Chen *et al.* [35] also found a similar change in the bandgap of the typical mixed perovskite (FAPbI_3_)_0.85_(MAPbBr_3_)_0.15_ under different strains. The measured ultraviolet (UV)-visible absorption spectra and PL spectra under tensile-strain, strain-free and compressive strain conditions demonstrated the reduction of bandgap with the increase of compressive strain (Fig. 6f). These strain-induced bandgap changes are consistent with the above first-principles calculations.

### Electrical properties

**Figure 7.**
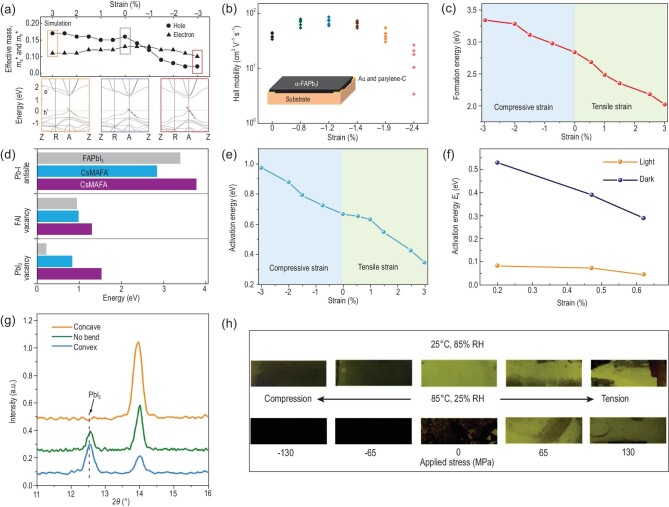
(a) Calculated effective masses of carriers of α-FAPbI_3_ perovskite under different strains from 3% to −3%. (b) Hole mobilities obtained by Hall effect measurements on α-FAPbI_3_ perovskite film [58]. Copyright 2020, Springer Nature. (c) Calculated strain-dependent formation energies of halide vacancies [57]. Copyright 2020, Springer Nature. (d) Calculated formation energies of antisites and Schottky vacancies in FAPbI_3_ and CsMAFA perovskites [51]. Copyright 2018, Springer Nature. (e) Calculated strain-dependent activation energies for the vacancy-assisted migration of halide ions [57]. Copyright 2020, Springer Nature. (f) The activation energy of ion migration of MAPbI_3_ films under different strains [34]. Copyright 2017, The American Association for the Advancement of Science. (g) Out-of-plane XRD patterns of the perovskite films under different external strains [34]. Copyright 2017, The American Association for the Advancement of Science. (h) Photographs of MAPbI_3_ film on PET substrate with externally applied stresses from −130 to 130 MPa aged at 25°C and 85% RH or 85°C and 25% RH for 24 h [31]. Copyright 2018, Wiley-VCH Verlag.

The strain-induced alteration of electronic band structures can further change the carrier dynamics of perovskites, since the effective mass of charge carriers is assessed by the band curvature extracted from first-principles calculations. Xu *et al.* [58] calculated the electron effective mass ($m_e^{\rm{*}}$) and hole effective mass ($m_h^{\rm{*}}$) under strain from 3% to −3%. Specifically, $m_h^{\rm{*}}$ is determined by the curvature of VBM, while $m_e^{\rm{*}}$ is from CBM. It has been discussed above that the VBM is more sensitive than CBM under strains. $m_e^{\rm{*}}$ therefore exhibits only a slight variation under strain between 3% and −3%, whereas compressive strain modulates the *E-k* dispersion of the valence band and greatly reduces the $m_h^{\rm{*}}$ (Fig. 7a). They further provided systematical characterizations of the effects of strain on the carrier dynamics of perovskites from experiment. Figure 7b shows the measured Hall effect carrier mobilities of the α-FAPbI_3_ films under strain from 0% to −2.4%. The films under −1.2% had the highest hole mobility, whereas further increasing the strain led to a drastic decrease of the hole mobility. This may be attributed to the higher dislocation densities arising from the high strain level. Figure S2c shows the calculated carrier mobility from time-of-flight measurements, which was plotted as a function of the strain applied. It exhibits a similar trend to the results measured from Hall effect. In addition to compressive strain, Figure S2d compares the hole mobilities of perovskite films with/without tensile strain measured by the space-charge-limited-current (SCLC) method [35], where tensile strain decreased the hole mobility, consistent with the calculated results under tensile strain as shown in Fig. 7a.

### Defect properties

Metal halide perovskites show extraordinary optoelectronic properties that are largely attributed to their unique electronic structure, where anti-bonding states (Pb 6s-I 5p coupling) are located at the VBM. This leads to the defect-tolerant properties of perovskites—shallow intrinsic defects in perovskites. This anti-bonding feature is in contrast with conventional semiconductors such as GaAs [64] and GaN [65] that possess a bonding VBM with deep defect states in the bandgap. However, the defects in perovskite films, especially at the film surface and grain boundaries, still induce trap states that dramatically impair both the efficiency and stability of PSCs [14]. The prevalence of intrinsic point defects including vacancies and interstitials correlates closely with their defect formation energy, which directly reflects the density of point defects in perovskites [66,67]. Several groups therefore calculated the formation energies of defects under different strain conditions using density functional theory (DFT). Our group compared the formation energies of halide vacancies under the strains from tensile to compressive. We found that the tensile strain decreases the formation energy of halide vacancies, whereas compressive strain increases their formation energies compared to strain-free perovskites (Fig. 7c) [57]. The low formation energy of defects under tensile strain results in the increase of non-radiative recombination, decreasing the device performance. Islam *et al.* [32] further calculated the thermodynamic transition levels for vacancy defects of FAPbI_3_ and FA_0.75_Cs_0.25_PbI_3_ under different strains. They revealed that higher pressures >2 GPa enable a shift of shallow to deep states of iodide vacancies in these perovskites that may act as non-radiative recombination centers, lowering the device efficiency. Fortunately, at low pressure <0.5 GPa, the transition state levels of these vacancies remain largely unchanged and would not significantly influence the lifetime of charge carriers.

The impact of local lattice strain on the defect properties of perovskites was recently reported by Sargent *et al.* [51]. They compared the calculated formation energies of antisites and vacancies in FAPbI_3_ and mixed CsMAFA perovskites including Cs_2_MA_12_FA_94_Pb_108_Br_55_I_269_ (CsMAFA′) and Cs_8_MA_12_FA_88_Pb_108_I_269_ (CsMAFA). It should be noted that the local lattice strain in FAPbI_3_ has been released by partially replacing FA and I with Cs/MA and Br ions. Although no notable difference was observed in the formation energy of Pb-I antisites, there was a significant difference in the formation energies of lead iodide vacancies (Fig. 7d). This energy in strained FAPbI_3_ is as low as ∼0.25 eV, while the value in non-strain CsMAFA is increased more than threefold. Therefore, the local lattice strain reduces the formation energies of lead iodide vacancies in perovskites.

### Ion migration

Ion migration has been reported to be one of the main causes of photocurrent hysteresis and instability in PSCs [68]. In contrast to the widely observed light-induced ion migration in perovskite films, the impact of strain on ion migration is just beginning to be explored. Our group recently calculated the relative activation energies (*E_a_*) for the vacancy-assisted migration of halide ions in perovskites—considering the lowest formation energy of halide vacancy—under biaxial strains from tensile to compressive (Fig. 7e). The activation energies for halide ion migration are 0.547, 0.667 and 0.794 eV for films under tensile strain (1.5%), no strain and compressive strain (−1.5%), respectively [57]. The above results indicate that compressive strain increases the activation energy, thereby decelerating the ion migration in perovskites [57,69].

Huang *et al.* [34] reported the first direct experimental evidence of activation energy (*E_a_*) for ion migration in perovskites, which quantitatively describes the degree of difficulty in ion migration. They deposited MAPbI_3_ films on flexible substrates, and then bent them to be concave or convex to introduce compressive or tensile strain. The temperature-dependent conductivity of the three types of films in the dark was measured. The activation energy for ion migration can be extracted through the Nernst-Einstein relationship:

(3) \begin{equation*}\sigma T = {\sigma _0}\ {\rm {exp}}\!\left( {\frac{{ - {E_a}}}{{kT}}} \right)\ ,\end{equation*}

where σ_0_ and *k* are the constants, σ and *T* are conductivity and temperature, respectively, and *E_a_* is the activation energy for ion migration. *E_a_* is derived from the slope of the ln(σ*T*) versus 1/*T* plot. As shown in Fig. 7f, the activation energies for ion migration in tensile-strain, non-strain and compressive-strain MAPbI_3_ films in the dark are 0.29, 0.39 and 0.53 eV, respectively. The values, under illumination by white light with an intensity of 25 mW cm^−2^, reduce to 0.046, 0.074 and 0.083 eV, respectively. The above measured activation energies demonstrate that perovskite films with tensile strain have smaller ion migration activation energy both in the dark or under illumination, whereas compressive strain increases the E_a_ for ion migration under the same conditions, consistent with our calculated results.

### Material stability

Point defects—particularly halide vacancies with the lowest formation energy—in perovskites have been regarded as a major source of instability of perovskites due to the following two reasons: (i) point defects have a high affinity for water and oxygen molecules, offering a facile path for oxygen into the perovskite lattice [51]; (ii) defects assist the migration of halide ions, leading to hysteresis in PSCs and halide segregation in mixed-halide perovskites, and subsequently phase segregation [70–73]. As discussed in the sections ‘Electrical properties’ and ‘Defect properties’, the formation energy of defects and activation energy for ion migration correlate closely with the strain in perovskites. The impact of strain in perovskites on their stability is therefore an important topic for the stability issues in perovskites.

Huang *et al.* [34] explored the connection between strain and the resulting photostability of perovskite film through depositing MAPbI_3_ film on flexible substrate. This flexible substrate can be conveniently used to apply external strains, from compressive to tensile, on perovskite films by bending them in a concave or convex shape. After illumination under white light with an intensity of ∼50 mW cm^−2^, they found that the convex film with a larger lattice strain had large yellow areas, indicating the decomposition of MAPbI_3_ into PbI_2_ as characterized by out-of-plane XRD; the concave film with the smallest strain remained mostly black without any appearance of the PbI_2_ peak (Fig. 7g). Therefore, the strain impact on the photostability of perovskite film is that tensile strain accelerates the degradation of perovskites under illumination.

Dauskardt *et al.* [31] carried out similar experiments to investigate the effect of strain on thermal and moisture stability of perovskites. MAPbI_3_ films deposited on polyethylene terephthalate (PET) with externally applied stresses ranging from −130 to 130 MPa were exposed to either damp air (25°C, 85% RH) or dry heat (85°C, 25% RH) for 24 h (Fig. 7h). They found that films with tensile stress exhibit moisture and thermal instability, as seen from the visible degradation with PbI_2_ formation, whereas films under compressive stress have an improved moisture and thermal stability. The improved illumination, moisture and thermal stability of perovskite films under compressive strain is attributed to the higher formation energies of point defects and higher activation energies for ion migration under compressive strain as discussed in the previous section.

## STRAIN-REGULATION METHODS FOR STABLE PEROVSKITES

As discussed above, strain affects physical properties of perovskites that consequently further influence the stability of perovskites. Therefore, regulating strain in perovskites is a novel and effective method to improve their intrinsic stability. In this section, we review recent efforts to regulate strain for stable perovskites. According to the different origins of strain, these approaches can be divided into two categories: (i) regulation of local strain; (ii) regulation of external condition-induced strain.

### Regulation of local strain

#### Incorporation of A/B/X-site ions

**Figure 8.**
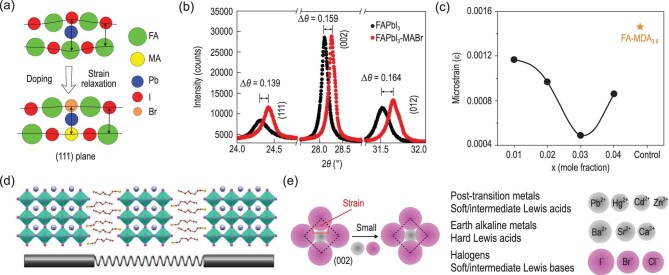
(a) Schematic illustration of the strain in (111) plane of FAPbI_3_, and strain relaxation after MABr alloying. (b) XRD patterns of FAPbI_3_ and FAPbI_3_-MABr [67]. Copyright 2016, The American Chemical Society. (c) Calculated strain in perovskite with a structure of FTO/mp-TiO_2_/perovskite [47]. Copyright 2020, The American Association for the Advancement of Science. (d) Schematic illustration of stress relaxation with soft and stiff structural subunits [74]. Copyright 2019, Wiley-VCH Verlag. (e) Schematic demonstrating strain reduction through incorporation of small B/X-site ions [51]. Copyright 2018, Springer Nature.

Compositional tuning of perovskites can be used to not only tune the bandgap by halide substitution on the X site, but also improve the thermal stability by Cs and FA substitution on the A site. Recently, new substitution-induced local strain relaxation phenomena have been observed. Priya *et al.* [67] revealed that the intrinsic instability mechanism of α-FAPbI_3_ is the existence of an anisotropic strained lattice in the (111) plane that drives phase transformation into the δ-phase (Fig. 8a). When FAPbI_3_ is alloyed with MABr (denoted as FAPbI_3_-MABr), the lattice size is reduced due to the smaller ionic radius of MA^+^ and Br^−^ compared to FA^+^ and I^−^, respectively. This substitution balances the lattice strain, leading to strain relaxation. The sharpening of (111) peaks in FAPbI_3_-MABr indicated the strain relaxation of the lattice (Fig. 8b), thereby stabilizing the α-phase of FAPbI_3_. Compared to pure FAPbI_3_ solar cells, the resultant FAPbI_3_-MABr devices exhibited largely improved stability, wherein no drop in efficiency was observed for the device kept under an RH of ∼50% for 1000 h (Fig. S3a). Seok *et al.* [47] further significantly lowered the lattice strain in FAPbI_3_ by introducing larger methylenediammonium (MDA^2+^) and smaller Cs^+^ together. They found that the alloyed FAPbI_3_ with 0.03 mol fraction of both MDA and Cs cations effectively reduced the local lattice strain as well as the trap density in PSCs (Fig. 8c). This led to the fabrication of PSCs with 24.2% and 21.6% certified efficiency for small and large (1 cm × 1 cm) devices. Moreover, the corresponding cells showed excellent thermal stability and maintained almost 80% of their initial efficiency in storage at 150°C for 20 h (Fig. S3b).

Similarly, Chen *et al.* [74] reported the stress relaxation in perovskite films via A site incorporation. They found that there exists inhomogeneous residual stress distribution across the film thickness direction, wherein the residual tensile stress in the top region of the perovskite film was remarkably higher than that at other depths of the film. This inhomogeneity was attributed to the gradient distribution of cation MA^+^ rather than Cs^+^, FA^+^, Br^−^ and I^−^. The surface residual stress was effectively released to ∼50% by introducing two kinds of A-site cations, octylammonium iodide (OAI) and phenethylammonium (PEAI), into the surface of the FA_0.85_MA_0.15_Pb(I_0.85_Br_0.15_)_3_ films. The mechanism for stress relaxation with OA treatment can be attributed to the incorporation of this large organic cation. This 2D perovskite component mainly dwells at the surface of films to construct soft structural subunits, providing extra structural flexibility to reduce the residual stress (Fig. 8d). The optimized device not only exhibited an improved efficiency of 21.48% (Fig. S3c), but also demonstrated excellent humidity stability and external stress endurance. The devices without encapsulation retained 95% of the initial efficiency after 1000 h under ambient conditions (Fig. S3d).

In addition to the above A/X-site incorporation, Sargent *et al.* [51] demonstrated the incorporation of judiciously selected B-site dopants into the lattice of mixed perovskite crystals that release the local lattice strain and then increase the formation energy of defects, resulting in the stability in ambient air being an order of magnitude higher. They chose Cd owing to its isovalence of Pb while having a smaller ionic radius (Fig. 8e). DFT calculated results indicated that Cd incorporation releases the lattice strain and suppresses the formation of I vacancy. The resultant unencapsulated Cd-containing CsMAFA PSCs thereby showed greatly improved stability. They retained >95% of their initial PCE after storage in the dark in ambient air for 30 days at a relative humidity of 50%, whereas CsFAMA PSCs maintained only 60% of their initial PCE (Fig. S3e). The operational MPP lifetime under the same conditions was also an order of magnitude longer than state-of-the-art CsMAFA perovskite solar cells (Fig. S3f). Additionally, the incorporation of Cl^−^ also decreased the lattice strain of CsMAFA perovskites due to the small ionic radius of Cl^−^, similar to the report by Priya *et al.* [67]. The Cl-containing CsMAFA films exhibited improved moisture and thermal stability, although the incorporation of additional Cl would increase the bandgap that is not desirable for solar cells.

#### Eliminating inhomogeneity in mixed halide perovskite films

**Figure 9.**
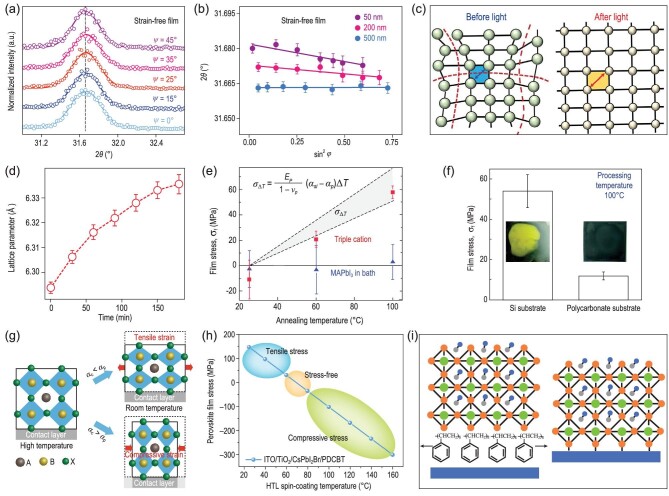
(a) GIXRD spectra at different tilt angles at a depth of 50 nm for the strain-free film. (b) Strain distribution at depths of 50, 200 and 500 nm for the strain-free film [35]. Copyright 2019, Springer Nature. (c) Schematic demonstrating the structure change before (local distortion) and after illumination (lattice expansion). (d) Illumination time-dependent lattice constant [75]. Copyright 2018, The American Association for the Advancement of Science. (e) Measured and calculated stress in MAPbI_3_ and CsMAFA films formed at different temperature. (f) Measured stress in MAPbI_3_ films formed at 100°C on Si and polycarbonate; insets show the photographs of MAPbI_3_ on Si and polycarbonate after 45 h of dry heat aging at 85°C and 25% RH [31]. Copyright 2018, Wiley-VCH Verlag. (g) Schematic demonstrating the formation of tensile and compressive strains. (h) The calculated PDCBT spin-coating temperature-dependent stress in perovskites within structures of ITO/TiO_2_/perovskite/PDCBT [57]. Copyright 2020, Springer Nature. (i) Schematic showing the mechanism of the PS buffer layer that releases the stress in perovskites [77]. Copyright 2019, Wiley-VCH Verlag.

Regarding the local lattice strain originated from the inhomogeneity in mixed halide perovskites, Chen *et al.* [35] adjusted the perovskite annealing process and proposed a flipped annealing method for the fabrication of perovskite films to modulate the gradient in-plane strain. Specifically, to eliminate the temperature gradient during perovskite film fabrication that induces the compositional inhomogeneity, they added a flipped annealing process to the conventionally annealed perovskite film. This treatment introduced compressive strain and released the tensile strain. GIXRD results showed that the tensile strain was significantly reduced in the sample through this treatment (Fig. 9a and b). Eventually, the optimized strain-free PSC reached a certified efficiency of 20.7% and an averaged efficiency of 19.8%, whereas tensile-strained devices only exhibited an efficiency averaging around 18.7% (Fig. S4a and b).

#### Lattice expansion-induced local strain relaxation

Light-induced structural dynamics have been demonstrated to play a vital role in the physical properties and stability of perovskites as well as their corresponding optoelectronic device performances. In addition to the widely observed light-induced ion migration and halide segregation, illumination can also lead to the relaxation of local strain in perovskites reported by Mohite *et al.* [75]. They found that continuous light soaking using a standard 1-sun (100 mW cm^−2^) source results in a large and uniform lattice expansion in perovskite films. They considered that photogenerated electron-hole pairs weaken covalent bonds, lead to either less-distorted Pb-I-Pb bonds or elongation of the Pb-I bonds, and finally cause lattice expansion, resulting in the relaxation of local lattice strain in perovskites (Fig. 9c). This expansion is especially observed in mixed-cation perovskites that are usually strained due to the distorted nature of the lattice with different size cations. Grazing-incidence wide-angle X-ray scattering (GIWAXS) measurements showed that all of the diffraction peaks shifted toward lower values of scattering vector q as the illumination time increased (Fig. S4c), corresponding to an isotropic increase in lattice constant (Fig. 9d)—so called lattice expansion. The sharpening of Bragg peaks further indicated the relaxation of local lattice strain. This light illumination treatment then increased the performance of p-i-n solar cells using NiO_x_ as HTL and a FA_0.7_MA_0.25_Cs_0.05_PbI_3_ active layer from 15% to 20.5% after 2 h of illumination (Fig. S4d). This improvement was attributed to the lattice expansion and a reduction in strain that lowered the energetic barriers at the perovskite-contact interfaces. Notably, the devices only exhibited a slow reduction in efficiency under continuous operation at 1-sun illumination for more than 1500 h (Fig. S4e). However, whether this is light-induced or heat-induced lattice expansion is still under debate. Dauskardt *et al.* [76] demonstrated that the mechanism for lattice expansion is heat-induced thermal expansion rather than light-induced expansion through controlling the temperature of perovskite films under both dark and illuminated conditions.

### Regulation of external condition-induced strain

As discussed in section ‘External condition-induced strain’, the external conditions that induce the strain in perovskites include two types of mismatches: thermal expansion and lattice mismatch between the perovskite and substrate. In this section, we mainly focus on the thermal expansion mismatch, which is the major origin of the strain in perovskite solar cells. With regard to the above-described correlation between stress and thermal expansion mismatch (see equation (1)), there have been several strain modulation strategies reported recently. These approaches can be divided into two categories: (i) lowering the formation temperature of perovskite films to reduce ΔT [31]; (ii) using contacting layers possessing similar α with perovskites to decrease Δα [34].

#### Lowering the processing temperature of perovskite films

Stress is built up during temperature changes, wherein higher annealing temperature results in larger stress values. In contrast, if a perovskite film is deposited at low temperature, even at room temperature, the stress would be decreased dramatically. Huang *et al.* [34] prepared MAPbI_3_ films at room temperature by drying the as-spun MAPbI_3_ · DMSO intermediate phase via evacuation for 3 days rather than annealing at 100°C. They found that the XRD peak positions of this film (pink line) and the non-strained crystals (green line) were nearly the same, indicating that the strain-free state in the perovskite film formed at room temperature (Fig. S5a). They further revealed that this strain-free MAPbI_3_ film was still unstrained after being heated at 100°C for 4 h, whereas the tensile-strain film was still strained even after being annealed at 100°C for 20 h, indicating that the strain state in perovskites after film formation is insensitive to post-annealing treatment. This can be attributed to the strong interaction between the perovskite and substrate once the perovskite is formed.

Dauskardt *et al.* [31] also reported that CsFAMA perovskite films that formed without any annealing (25°C) exhibited a stress of −10.8 ± 15.2 MPa, whereas films annealed at 60 and 100°C resulted in tensile stresses of 20.7 ± 6.6 and 57.6 ± 4.9 MPa, respectively (Fig. 9e). These results indicate that the tensile strain in perovskite films can be reduced by lowering the formation temperature of perovskites. They further fabricated perovskite solar cells with the above CsFAMA films in a p-i-n architecture by employing polytriarylamine (PTAA) as the HTL and C_60_ as the ETL. Figure S5b shows the efficiency of these CsMAFA devices formed at 25, 60 and 100°C, indicating an improvement in PCE with annealing temperature. Although lowering the annealing temperature has been proven to be an efficient way to diminish the tensile stress in perovskite films, this strategy also lowers the device efficiency, which can be attributed to the lower quality of perovskite films fabricated by low-temperature processing.

#### Using contacting layers with high thermal expansion coefficients

According to equation (1), lowering the thermal expansion difference between the perovskite and contacting layer (Δα) is another way to reduce the tensile strain in perovskites [57]. PSCs typically consist of a stack of multilayers including a substrate with a transparent conducting oxide electrode layer, followed by the ETL, perovskite layer, HTL and another electrode layer. The contacting layers of perovskites include substrate and charge-transport layers that usually possess different thermal expansion coefficients (Fig. 3a). Therefore, thermally-induced stress in perovskites can be regulated by choosing suitable contacting layers with similar thermal expansion coefficients to that of perovskites.

Huang *et al.* [34] deposited MAPbI_3_ film on a flexible substrate of PET that had a thermal expansion coefficient of 2 × 10^−5^ to 8 × 10^−5^ K^−1^, close to that of MAPbI_3_. The out-of-plane XRD measurements showed that the XRD peaks of the film on PET shifted to lower angles compared to those on ITO/glass (Fig. S5c). This shift indicated that the tensile strain in the MAPbI_3_ film on PET substrate was much smaller than that on ITO/glass substrate. Dauskardt *et al.* [31] compared the stress in MAPbI_3_ films formed at 100°C on Si with a low thermal coefficient of 2.6 × 10^−5^ K^−1^ and polycarbonate with a high thermal coefficient of 2 × 10^−5^ K^−1^. As shown in Fig. 9f, the average stresses are 52 and 12 MPa in the films deposited on Si and polycarbonate substrate, respectively. Therefore, the utility of flexible substrates with high α can significantly reduce the thermally induced tensile stress in perovskite films.

Our group [57] recently reported a strain-compensation strategy that reduces the tensile strain in perovskite films by introducing an external strain from the HTL. The keys for this strain-compensation strategy are the following three aspects. (i) The top HTL layer should have a higher thermal expansion coefficient compared with the perovskite, offering the possibility of compressive strain (Fig. 9g). The poly[5,5-bis(2-butyloctyl)-(2,2-bithiophene)-4,4′-dicarboxylate-alt-5,5′-2,2′-bithiophene] (PDCBT) with a similar chemical structure to poly(3-hexylthiophene-2,5-diyl) (P3HT) is chosen due to its high thermal expansion coefficient (31.5 × 10^−5^ K^−1^). (ii) The functional layer should have a strong interaction with the perovskite in order to anchor to the lattice and achieve strain offset. The X-ray photoelectron spectroscopy (XPS) results showed that strong interaction exists between the perovskite and PDCBT through the formation of Pb-O bonds. (iii) The top interface layer should be coated at high temperatures, inducing a compressive strain when cooling back to room temperature (Fig. 9h). Finally, when coating PDCBT at high temperatures, the XRD peaks of as-fabricated perovskite film shifted to higher diffraction angles compared to that of scraped perovskite powder (Fig. S5d). This indicated that the residual tensile strain in perovskite film is successfully compensated through depositing the HTL atop the perovskite at high temperature to introduce compressive strain [57].

Our group further fabricated three types of PSCs based on different strain-state perovskite films—tensile strain, strain free and compressive strain—for the processing temperatures of the PDCBT HTL coated at 60, 90 and 120°C, with a planar architecture of ITO/TiO_2_-Cl/perovskite/PDCBT/MoO_x_/Au. Despite the comparable performance of PSCs under different strains, the photostability under maximum power point (MPP) conditions and thermal stability of PSCs under continuous heating at 85°C varied greatly with strain (Fig. S5e and f). The compressive-strain device maintained 95% and 96% of its initial PCEs after continuous MPP operation for 60 and 1000 h of heating at 85°C, respectively, greatly surpassing the strain-free and tensile-strain devices [57].

Another stress-regulation strategy is the direct isolation of the perovskite and substrate, enabled by their mismatched thermal expansion coefficients, and then the addition of a soft buffer layer between them. Meng *et al.* [77] recently introduced polystyrene (PS) into the PSCs as the buffer layer between the SnO_2_ and perovskite (Fig. 9i). The GIXRD results showed that residual stresses exist in both the control film (SnO_2_/perovskite) and experimental film (SnO_2_/PS/perovskite) as evidenced by the gradual shift of the peaks at 14.0 degrees (Fig. S6a and b); however, the PS-treated film exhibits a smaller slope than the control film, indicating less residual stress in the SnO_2_/PS/perovskite (Fig. S6c). This revealed that the addition of a PS layer can release the thermally induced residual stress in perovskite due to the soft feature of PS with a low glass transition temperature. The resultant strain-free PS-modified PSCs achieved a high efficiency of 21.89% and retained almost 90% and 97% of their initial PCEs after continuous MPP operation for 72 h and after 5 days of ‘day cycle’ stability test, respectively (Fig. S6d).

## SUMMARY AND OUTLOOK

**Figure 10.**
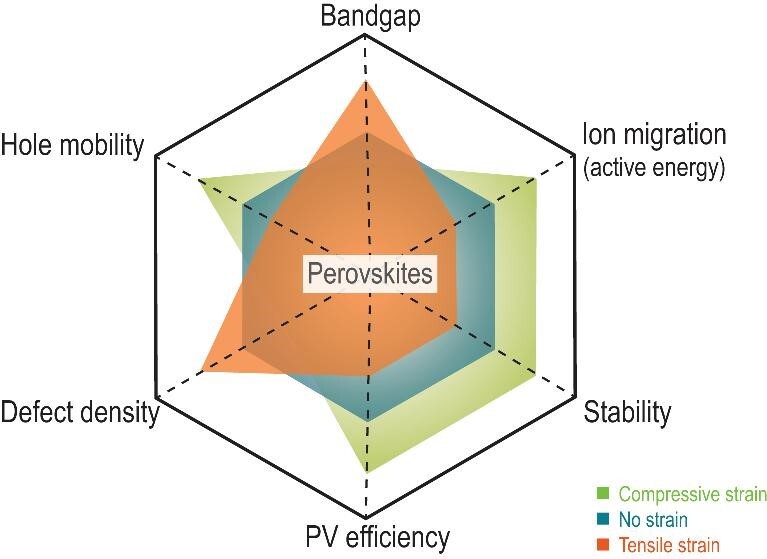
Summary of the impacts of various strains on perovskite and device performance.

We herein presented an overview of the origin, impact and regulation of the strain in perovskites, which govern the performance and stability of perovskite solar cells. The strain in perovskites mainly originates from the local lattice strain and external condition-induced strain, including the thermal expansion and lattice mismatch between the perovskite and substrate. The impacts of strain on the physical properties of perovskites, including bandgap, ion migration, hole mobility, defect density and stability, as well as perovskite-based PV efficiency, are complex. For example, the intrinsic interplay between the defect and strain is ambiguous—whether strain induces the generation of defects, or vice versa. Further investigation is needed to uncover the relationship between strain and its impacts. In addition, shear strain that should be more obvious in large area panels and flexible devices is still rarely investigated, which may be a significant topic for future research. Figure 10 presents the confirmed relationship between strain and these properties. The key impact of strain on perovskites is that tensile strain accelerates the degradation of perovskites and associated PV devices; compressive strain improves the intrinsic stability of perovskites and their corresponding PV devices. The aim of strain regulation in perovskites is therefore to release tensile strain and introduce compressive strain. Strain engineering in perovskite films has recently emerged as a central front in advancing perovskite-based optoelectronic device stability and performance.

Despite several years of extensive research on the strain in perovskites, much room remains to further advance the concepts reviewed herein. Although incorporation of A/B/X-site ions is an effective method to relax local strain and increase material stability, this may change the physical properties of perovskites and decrease the device efficiency. Whether light or heat induces the lattice expansion for strain relaxation is still under debate. Other strain regulation methods, such as low-temperature fabrication of perovskite films and utilization of flexible substrate, can diminish tensile strain in perovskite films but lower the device performance. The following strategies may be of great importance for further enhancing the stability and performance of perovskite solar cells through strain engineering: (i) design of mesoporous substrate for the deposition of the perovskite layer such as mesoporous TiO_2_ and ZrO_2_, wherein the mesoporous layer can extrinsically shield the strain in perovskite layers due to the disruption of the continuity of perovskite films [78]; (ii) exploration of new substrates possessing similar thermal expansion coefficients as perovskites—the currently utilized flexible substrates are limited to PET and polycarbonate; (iii) based on equation (1), one of the methods to decrease tensile strain is to reduce Δα—considering the low thermal expansion coefficients of widely used substrates such as ITO-coated glass, decreasing the thermal expansion coefficient of perovskites may be another way to reduce the detrimental tensile strain in perovskite films; (iv) besides the reported external compressive strain from hole-transport layer, other functional layers can also offer compressive strain for perovskites, for example, encapsulant layers, especially encapsulant polymers, should not only block air and moisture but also provide compressive strain due to their high thermal expansion coefficients and high processing temperature—many encapsulants usually require processing at temperatures of ∼150°C to cross-link the material and then adhere well to PSCs; (v) exploration of new synthesis methods to fabricate high-quality perovskite films under low processing temperature. Considering that strain regulation is just emerging, and requires more knowledge on origins, impacts and engineering, strain engineering in perovskites will continue to help drive the higher performance of perovskite solar cells.

## Supplementary Material

nwab047_Supplemental_File
