# Supplementary material for: Strain in perovskite solar cells: origins, impacts and regulation
Source: Natl Sci Rev. 2021 Mar 23;8(8):nwab047. doi: 10.1093/nsr/nwab047 (PMC8363326; doi:10.1093/nsr/nwab047)
Supplement: nwab047_Supplemental_File [file nwab047_supplemental_file.docx]

Supplementary Data

Strain in perovskite solar cells: origins, impacts, and regulation

Jinpeng Wu^1,3,†^, Shun-Chang Liu^1,3,†^, Zongbao Li^2^, Shuo Wang^1^, Ding-Jiang Xue^1,3,^*, Yuan Lin ^1,3,^* and Jin-Song Hu^1,3,^*

1Beijing National Laboratory for Molecular Sciences (BNLMS), Institute of Chemistry, Chinese Academy of Sciences, Beijing 100190, China

2School of Material and Chemical Engineering, Tongren University, Tongren 554300, China

3School of Chemical Sciences, University of Chinese Academy of Sciences, Beijing 100049, China

* Corresponding authors. E-mails: djxue@iccas.ac.cn; linyuan@iccas.ac.cn; hujs@iccas.ac.cn

† Equally contributed to this work


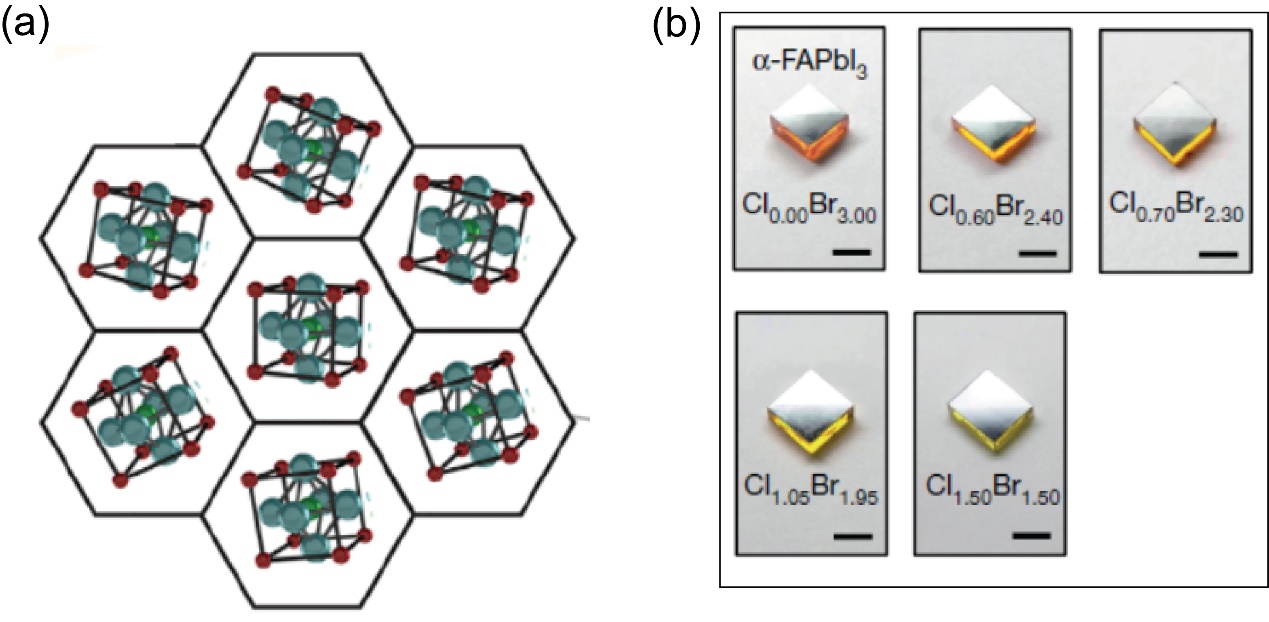


**Figure S1.** (a) Schematic illustration of sub-grain orientation heterogeneity induced stain in CH_3_NH_3_PbI_3_ perovskite films [52]. Copyright 2019, The Elsevier. (b) Optical images of as-prepared epitaxial α-FAPbI_3_ films. Scale bars, 4 mm [58]. Copyright 2020, The Springer Nature.


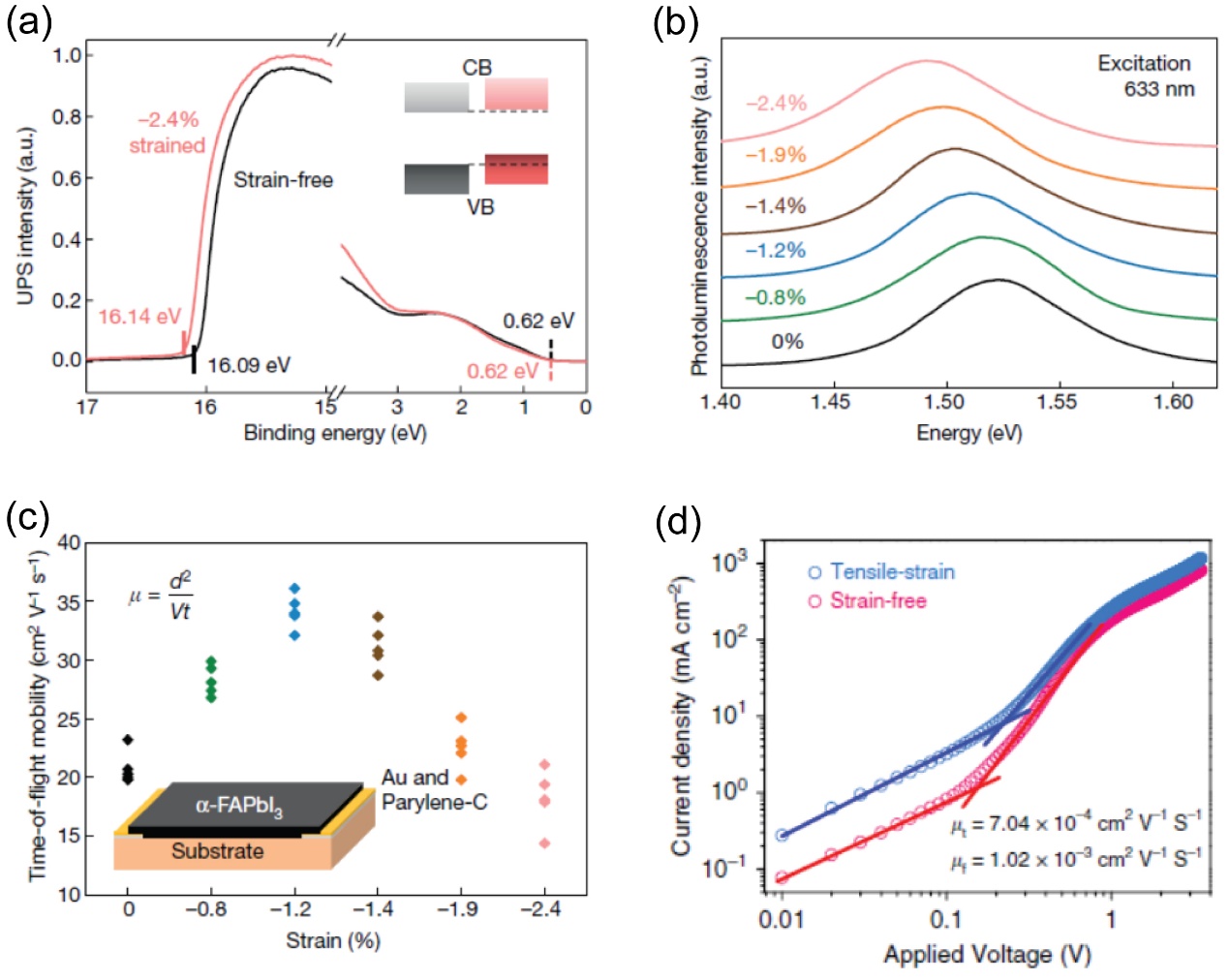


**Figure S2.** (a) UPS spectra of a -2.4% strained and a non-strained sample. (b) PL spectra of α-FAPbI_3_ films under gradually increased compressive strains. (c) Plots of calculated carrier mobilities from time-of-flight measurements on α-FAPbI_3_ perovskite film as a function of the strain magnitudes [58]. Copyright 2020, The Springer Nature. (d) Current-voltage curves of the hole only SCLC device of (FAPbI_3_)_0.85_(MAPbBr_3_)_0.15_ perovskite film with/without tensile strain [35]. Copyright 2019, The Springer Nature.


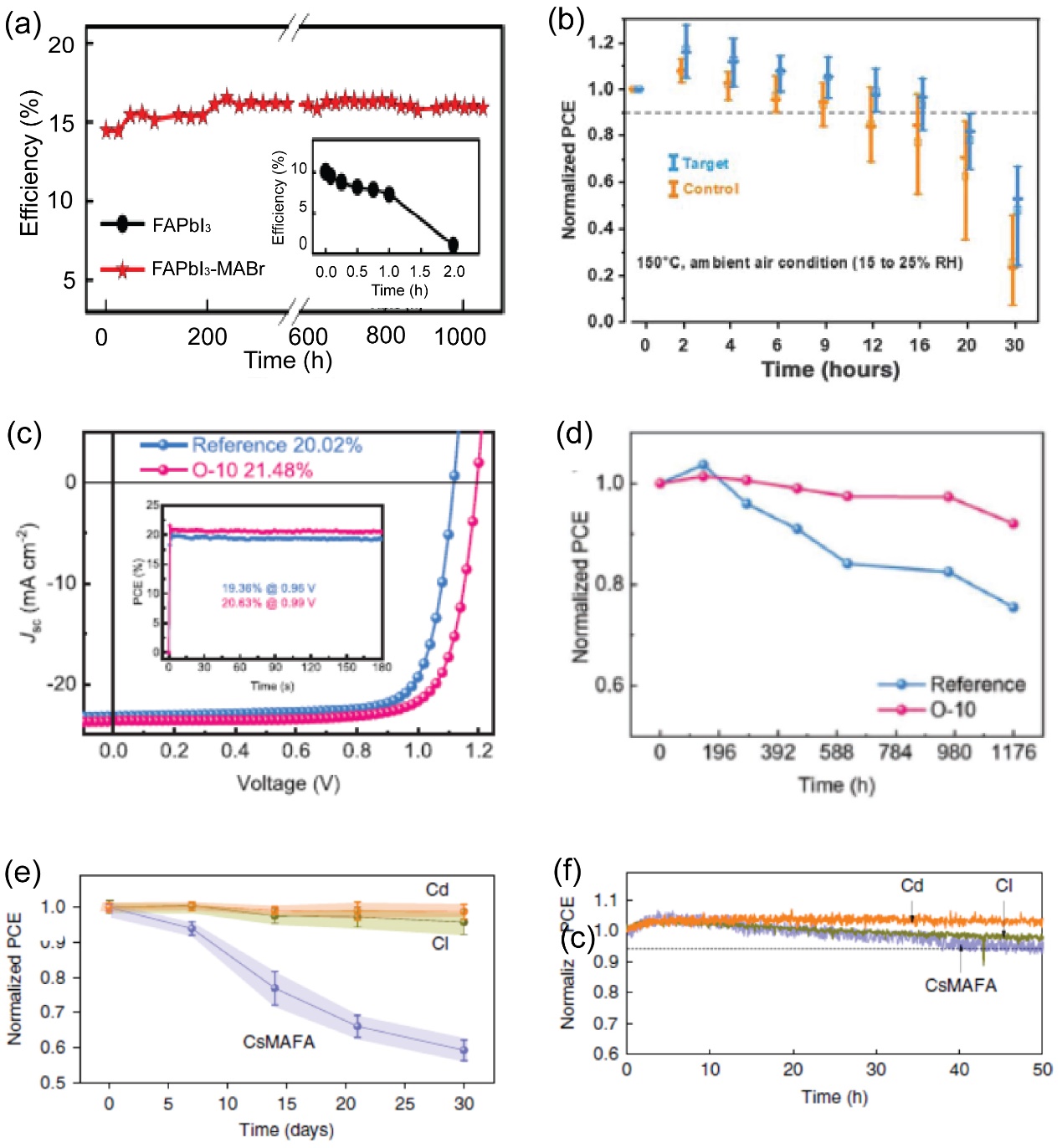


**Figure S3.** (a) Evolution of PCE of solar cells based on FAPbI_3_ (inset) and FAPbI_3_-MABr aged in air under a RH of ~50% at room temperature without encapsulation [67]. Copyright 2016, The American Chemical Society. (b) Comparison of thermal stability at 150 ^o^C of unencapsulated control and target PSCs [47]. Copyright 2020, The American Association for the Advancement of Science. (c) J-V curves of the best-performance devices based on the reference and O-10 perovskite films; the inset shows the evolution of PCEs of the champion device under MPP tracking. (d) Evolution of PCEs of PSCs stored in air with humidity 16-50% for over 1000 h without encapsulation [74]. Copyright 2019, The Wiley-Blackwell. (e) Evolution of PCEs of solar cells aged in ambient air. (f) Evolution of normalized PCEs under MPP tracking in a nitrogen atmosphere [51]. Copyright 2018, The Springer Nature.


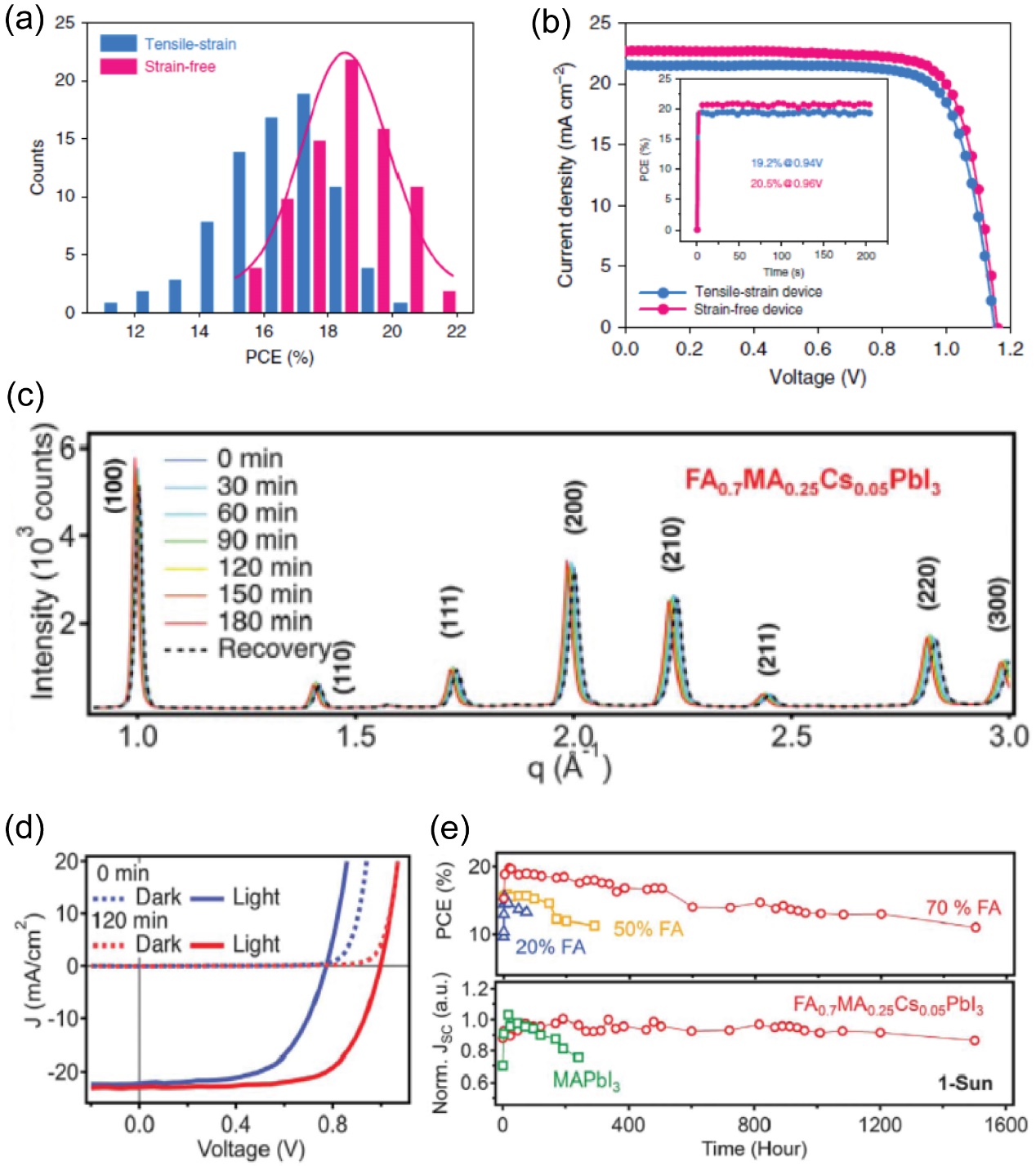


**Figure S4.** (a) Histograms of the PCEs for the devices with different strain. (b) J-V curves of the device with tensile strain and strain-free conditions [35]. Copyright 2019, The Springer Nature. (c) Line cut of GIWAXS maps for FA_0.7_MA_0.25_Cs_0.05_PbI_3_ films under different illumination times [72]. Copyright 2018, The American Association for the Advancement of Science. (d) J-V curves of device in the dark and under AM1.5G solar simulator illumination. (e) Evolution of performance (PCE and J_sc_) of devices under constant 1-sun illumination [75]. Copyright 2018, The American Association for the Advancement of Science.


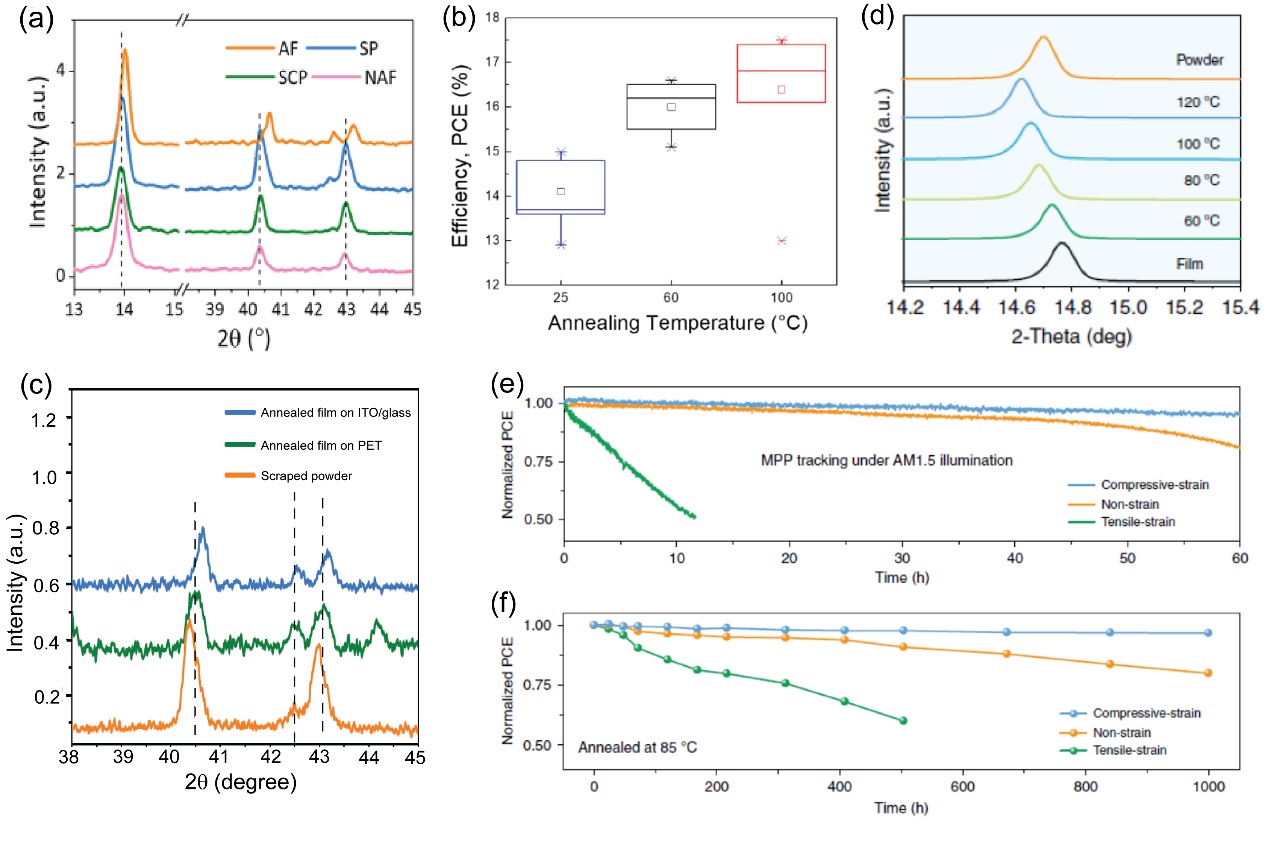


**Figure S5.** (a) Out-of-plane XRD patterns of the MAPbI_3_ annealed film (AF), Scraped powder (SP), single-crystal powder (SCP), and non-annealed film (NAF) [34]. Copyright 2017, The American Association for the Advancement of Science. (b) PCE statistics of CsMAFA devices formed at 25 ^o^C, 60 ^o^C, and 100 ^o^C [31]. Copyright 2018, Wiley-VCH Verlag. (c) Out-of-plane XRD patterns of the MAPbI_3_ scraped powder, annealed films on ITO/Glass and PET substrates [34]. Copyright 2017, The American Association for the Advancement of Science. (d) Magnified (100) diffraction peak patterns of perovskite powder, film and perovskite/PDCBT films deposited at different PDCBT spin-coating temperatures. (e) Evolution of PCEs under MPP tracking and continuous illumination. (f) Evolution of PCEs stored at 85 ^o^C in a nitrogen atmosphere [57]. Copyright 2020, The Springer Nature.


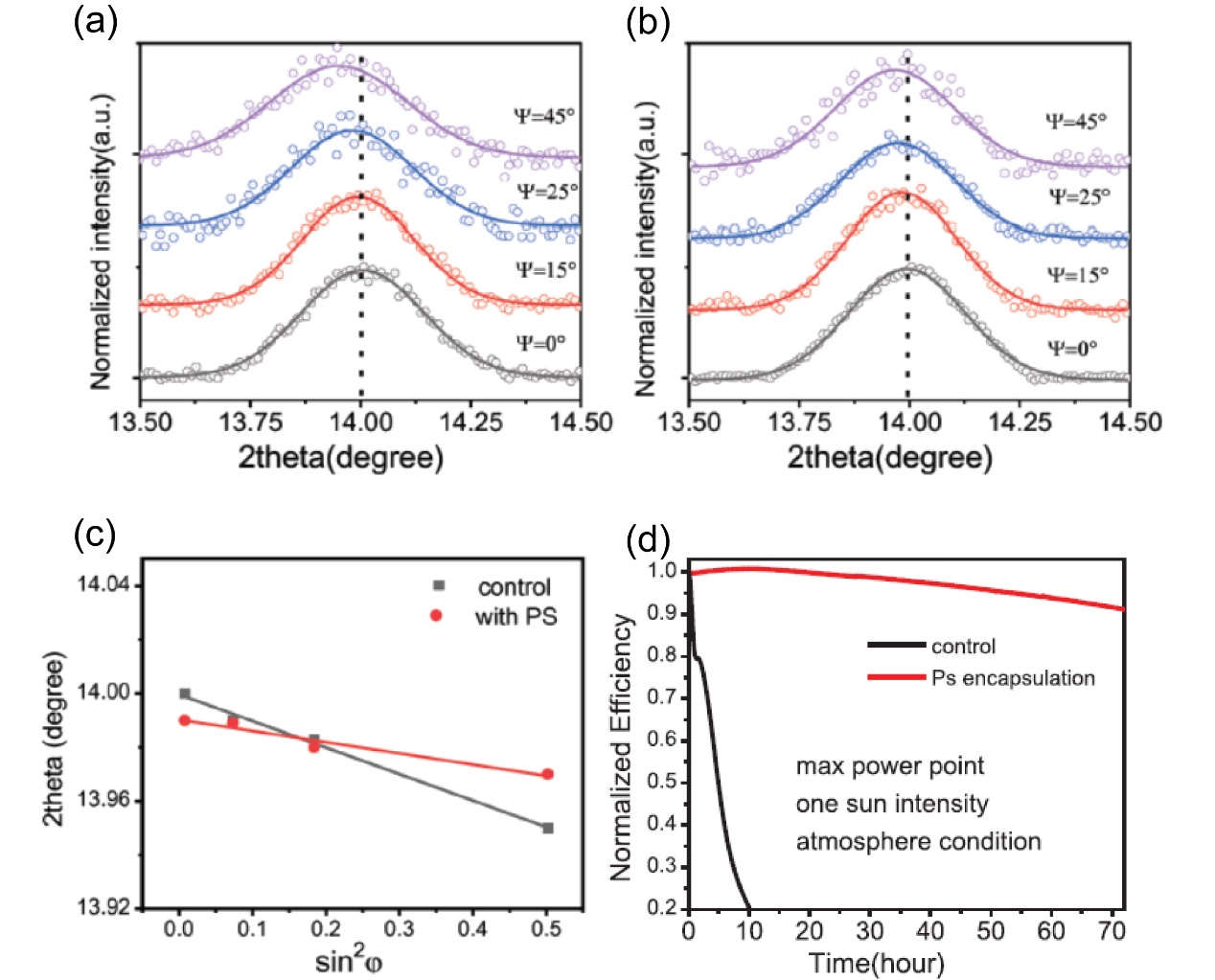


**Figure S6.** GIXRD patterns of (a) ITO/SnO_2_/PVSK film (control) and (b) ITO/SnO_2_/PS/PVSK film (with PS film) at different tilt angles. (c) Residual stress distribution in control and PS-modified perovskite films. (d) Evolution of PCEs of control and PS-encapsulated devices under MPP tracking [77]. Copyright 2019, Wiley-VCH Verlag.
